# Editorial: Prognostic factors in pituitary tumors: clinical, biochemical, imaging, and pathological aspects

**DOI:** 10.3389/fendo.2024.1492448

**Published:** 2024-10-23

**Authors:** Toru Tateno, Roxana-Ioana Dumitriu Stan, Stephanie Du Four

**Affiliations:** ^1^ Department of Medicine, University of Alberta, Edmonton, AB, Canada; ^2^ Department of Endocrinology, ‘Carol Davila’ University of Medicine and Pharmacy, Bucharest, Romania; ^3^ Department of Neurosurgery, University Hospital Brussels, Brussels, Belgium

**Keywords:** pituitary tumors, prognostic factors, complications - pituitary surgery, gene expression, biomarkers

Pituitary tumors, affecting nearly 17 percent of the population, carry substantial implications (1). While functional pituitary tumors can be effectively monitored for recurrence using hormonal biomarkers, the absence of an equivalent parameter for predicting recurrence in non-functional pituitary tumors results in delayed recognition and subsequent management. Additionally, there remains limited data regarding the risk of complications following transsphenoidal surgery for pituitary tumors. Consequently, there exists a critical need to identify prognostic biomarkers that can serve as surveillance tools for patients and potential therapeutic targets. The recognition of prognostic factors in pituitary tumors holds significant promise for tailoring personalized therapeutic approaches. By doing so, we can enhance treatment success rates, improve survival outcomes, and elevate overall quality of life for affected patients. This Research Topic comprises three original research articles, one case report with a literature review, and one comprehensive review, all aimed at advancing our understanding of prognostic factors in pituitary tumors.

One subtype of aggressive pituitary tumors is the plurihormonal Pit1-lineage pituitary tumor (2–4). However, due to its rarity, clinical features associated with GH/TSH-cosecreting pituitary tumors remain scarce. Consequently, managing this specific tumor type poses significant challenges. Yu et al. retrospectively reviewed 12 GH/TSH-cosecreting pituitary tumor from 2063 patients diagnosed with GH-secreting pituitary tumorshen. GH/TSH-cosecreting pituitary tumors are more aggressive and resistant to therapies. Also, higher occurrence rates of complications, such as arrhythmia, heart enlargement, and osteoporosis/osteopenia, were seen in GH/TSH-cosecreting pituitary tumors than GH-secreting pituitary tumors clinical features of GH/TSH-cosecreting pituitary tumors.

Regarding aggressive prolactinomas, they show resistance to multimodal therapies (5). Medina et al. reported a case with aggressive prolactinoma treated with a potent and selective multi-targeted receptor tyrosine kinase inhibitor, pazopanib with a literature review regarding potential biomarkers for aggressive prolactinomas. The literature review suggests potential risk factors for aggressive prolactinomas, including genetic and epigenetic changes as well as receptor expression profiles. The article shed the light into the pathological molecular findings in aggressive pituitary tumors, which ca be candidate molecules for personalized medicine in the future.

Bioinformatics is an integral discipline in cancer research that utilizes advanced computational strategies to decipher complex biological data sets. This approach will facilitate advances in precision medicine by greatly increasing our understanding of the molecular complexities underlying cancer and facilitating the design of treatments tailored to an individual’s genetic makeup. Chen et al. conducted a bioinformatics analysis using the Gene Expression Omnibus database, which revealed the correlation between the expression of basement membrane genes, including SPARCL1, GPC3, LAMA1, SDC4, GPC4, ADAMTS8, LAMA2, LAMC3, SMOC1, LUM and THBS2 genes, and immune infiltration. Based on the findings, a novel risk model, which predicts the invasiveness of pituitary tumors, has been proposed. These findings may lead to close surveillance of patients with pituitary tumors which have high risk of aggressive features.

One of the complications of transsphenoidal surgery for pituitary tumors is cerebrospinal fluid (CSF) leakage. The incidence of CSF leakage has been reported between 0.5%-15.0% (6–8). CSF leak is often associated with various complications and may increase the long-term and cost of hospitalization and affect the prognosis of patients. Therefore, identification of the prognostic factors of postoperative CSF leakage is crucial for early intervention. Previous studies suggest several factors, including BMI, tumor invasiveness, intraoperative cerebrospinal fluid leakage as the risk factors. Currently, there remains no consensus or comprehensive agreement regarding the risk factors associated with CSF leakage following transsphenoidal surgery for pituitary tumors. Zhao et al. conducted a systematic review and meta-analysis, examining risk factors associated with postoperative cerebrospinal fluid (CSF) leakage in patients with pituitary tumors. Their study included 6775 patients across eighteen articles. The identified risk factors encompassed body mass index (BMI), history of multiple operations, tumor size, tumor invasion, and tumor hard texture. The findings will lead to early medical interventions for CSF leakage.

Another complication of post transsphenoidal surgery is hyponatremia with incidence between 6.3% and 23. 4%, which may be symptomatic or asymptomatic. Previous studies suggested risk factors of hyponatremia after transsphenoidal surgery, however, they have been still debatable. Lin et al. reported a new nomogram based on the multivariable model of risk factors, such as findings regarding pituitary stalk and diaphragm sellae on MRI scan, postoperative diabetes insipidus, blood sodium levels on the second day after surgery, to predict the risk of delayed hyponatremia after transsphenoidal surgery. The novel nomogram achieved an AUC of 0.849, which can be used by neurosurgeons in a clinical setting to provide more accurate predictions of delayed hyponatremia after transsphenoidal surgery in patients with pituitary tumors.

Collectively, the articles featured in this Research Topic contribute novel evidence regarding prognostic factors in patients with pituitary tumors. Through comprehensive analyses of clinical, radiological, pathological, molecular, and bioinformatic data, these studies identify innovative prognostic markers. These markers hold promise for predicting both the prognosis of pituitary tumors and complications associated with transsphenoidal surgery, ultimately benefiting patients. However, further studies are necessary to validate these findings.
